# Differential Proliferation Rhythm of Neural Progenitor and Oligodendrocyte Precursor Cells in the Young Adult Hippocampus

**DOI:** 10.1371/journal.pone.0027628

**Published:** 2011-11-14

**Authors:** Yoko Matsumoto, Yuji Tsunekawa, Tadashi Nomura, Fumikazu Suto, Miho Matsumata, Shigeru Tsuchiya, Noriko Osumi

**Affiliations:** 1 Division of Developmental Neuroscience, Center for Translational and Advanced Animal Research, Graduate School of Medicine, Tohoku University, Sendai, Japan; 2 Department of Pediatrics, Tohoku University Hospital, Sendai, Japan; 3 Department of Biology, Kyoto Prefectural University of Medicine, Kyoto, Japan; 4 Department of Ultrastructural Research, National Institute of Neuroscience, National Center of Neurology and Psychiatry, Tokyo, Japan; 5 Department of Developmental Gene Regulation, Brain Science of Institute, RIKEN, Wako, Japan; Rikagaku Kenkyūsho Brain Science Institute, Japan

## Abstract

Oligodendrocyte precursor cells (OPCs) are a unique type of glial cells that function as oligodendrocyte progenitors while constantly proliferating in the normal condition from rodents to humans. However, the functional roles they play in the adult brain are largely unknown. In this study, we focus on the manner of OPC proliferation in the hippocampus of the young adult mice. Here we report that there are oscillatory dynamics in OPC proliferation that differ from neurogenesis in the subgranular zone (SGZ); the former showed S-phase and M-phase peaks in the resting and active periods, respectively, while the latter only exhibited M-phase peak in the active period. There is coincidence between different modes of proliferation and expression of cyclin proteins that are crucial for cell cycle; cyclin D1 is expressed in OPCs, while cyclin D2 is observed in neural stem cells. Similar to neurogenesis, the proliferation of hippocampal OPCs was enhanced by voluntary exercise that leads to an increase in neuronal activity in the hippocampus. These data suggest an intriguing control of OPC proliferation in the hippocampus.

## Introduction

In the mammalian hippocampus, persistent neurogenesis is prominent exclusively in the subgranular zone (SGZ) of the dentate gyrus (DG), which is important for hippocampus-dependent memory consolidation [1], [2]. Oligodendrocyte precursor cells (OPCs) are another persistent cycling cells that distributed throughout the adult rodent brains [3], [4], [5]. OPCs comprise ∼5% of all cells in the adult rodent brain [3], [6] and have been thought as a constitutive reservoir of oligodendrocytes that replace damaged myelin [5] or add de novo myelination [7]. However, they themselves appear to have potential to be constituents of neural circuits [8], [9], receiving synaptic inputs in the hippocampus [10]. A further mechanism involving communication between neurons and OPCs can be envisaged by observing their proliferating manner. During division OPCs maintain their morphological and physiological features, such as radial branched processes and synaptic responses in the hippocampus [11], [12].

Rhythmicity in biological activities is a common trait in a diverse range of organisms from prokaryotes to humans [13]. In a variety of mammalian organs, cell-cycle progression is under the control of circadian oscillatory mechanisms [14], [15], and disruption of clock-associated genes significantly affects genomic replication and cell division in regenerated tissues and tumors [16]. The division of neural stem/progenitor cells in the hippocampal neurogenic area is controlled by time-of-day-regulated mechanisms which may dictate daily modifications of dentate gyrus physiology [17]. The production of cells at proper timing would be essential for sustaining the housekeeping functions of tissues and organs.

In the adult hippocampus, the biological property of neurogenesis, including its rhythmicity, has been well studied [17], [18], [19], but the proliferation characteristics of OPCs remain uncertain. Here we explore features of OPC proliferation in the normal healthy condition of the hippocampus as compared with neurogenesis.

## Results

### Identification of proliferating cells in the hippocampus

**Figure 1:**
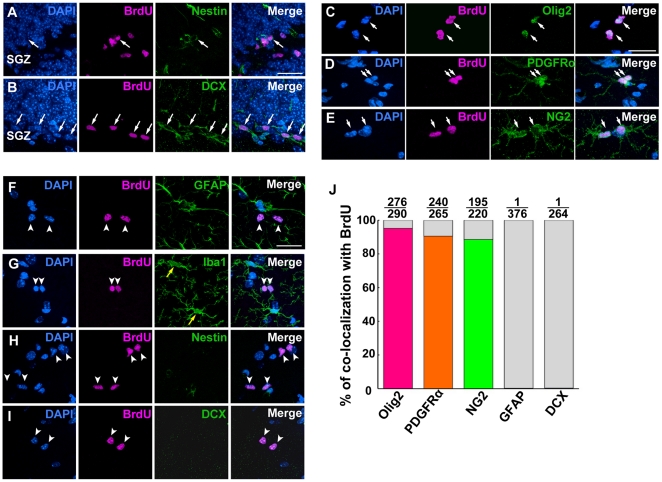
Analyses of cell-type specific markers in BrdU-positive cells in the hippocampal neurogenic area and non-neurogenic area. (A, B) Immunostaining with anti-BrdU, anti-nestin (A), and anti-DCX (B) antibodies in the neurogenic area. White arrows indicate BrdU-positive cells labeled with neural stem/progenitor cell markers. (C to E) Double immunostaining with anti-BrdU and anti-Olig2 (C), anti-PDGFRα (D), or anti-NG2 (E) antibodies in the non-neurogenic area. White arrows indicate BrdU-positive cells labeled with each OPC marker (C to E). Note that the dividing OPCs extend processes. (F to I) Double immunostaining with anti-BrdU and anti-GFAP (F), anti-Iba1 (G), anti-nestin (H) and anti-DCX (I) antibodies in the non-neurogenic area. White arrowheads indicate BrdU-positive cells that are negative for GFAP (F), Iba1 (G), nestin (H), and DCX (I). Yellow arrows indicate Iba1-positive cells (G). (J) The percentage of cells positive for each marker among all BrdU-positive cells. Scale bars: 20 µm (A to I).

We characterized the types of proliferating cells in the hippocampus by the immunohistochemical analyses using BrdU, a thymidine analog that labels S-phase cells. In the neurogenic area (Figure S1), i.e., the subgranular zone (SGZ) of the hippocampal dentate gyrus (DG), a large number of cells expressing nestin, a neural stem/progenitor marker and DCX, an immature neuronal marker were observed in BrdU-positive cells (Figure 1A, B). There were no BrdU-labeled cells that were positive for PDGFRα, an OPC marker in the SGZ. In the non-neurogenic area of the hippocampal gray matter (Figure S1), however, the majority of BrdU-labeled cells were positive for markers of OPCs such as Olig2 (95.2%, Figure 1C, J), PDGFRα (90.6%, Figure 1D, J) and NG2 (88.6%, Figure 1E, J). With regard to markers for other cell types, an astrocyte marker GFAP and a microglia marker Iba1 were not observed in the BrdU-positive cells (Figure 1F, G, J). Neither nestin nor DCX was observed in BrdU-positive cells localized in the non-neurogenic area (Figure 1H, I, J). These results suggest that OPCs are the major proliferating cell type in the non-neurogenic hippocampal area.

### OPCs show synchronized proliferation in the hippocampus

**Figure 2:**
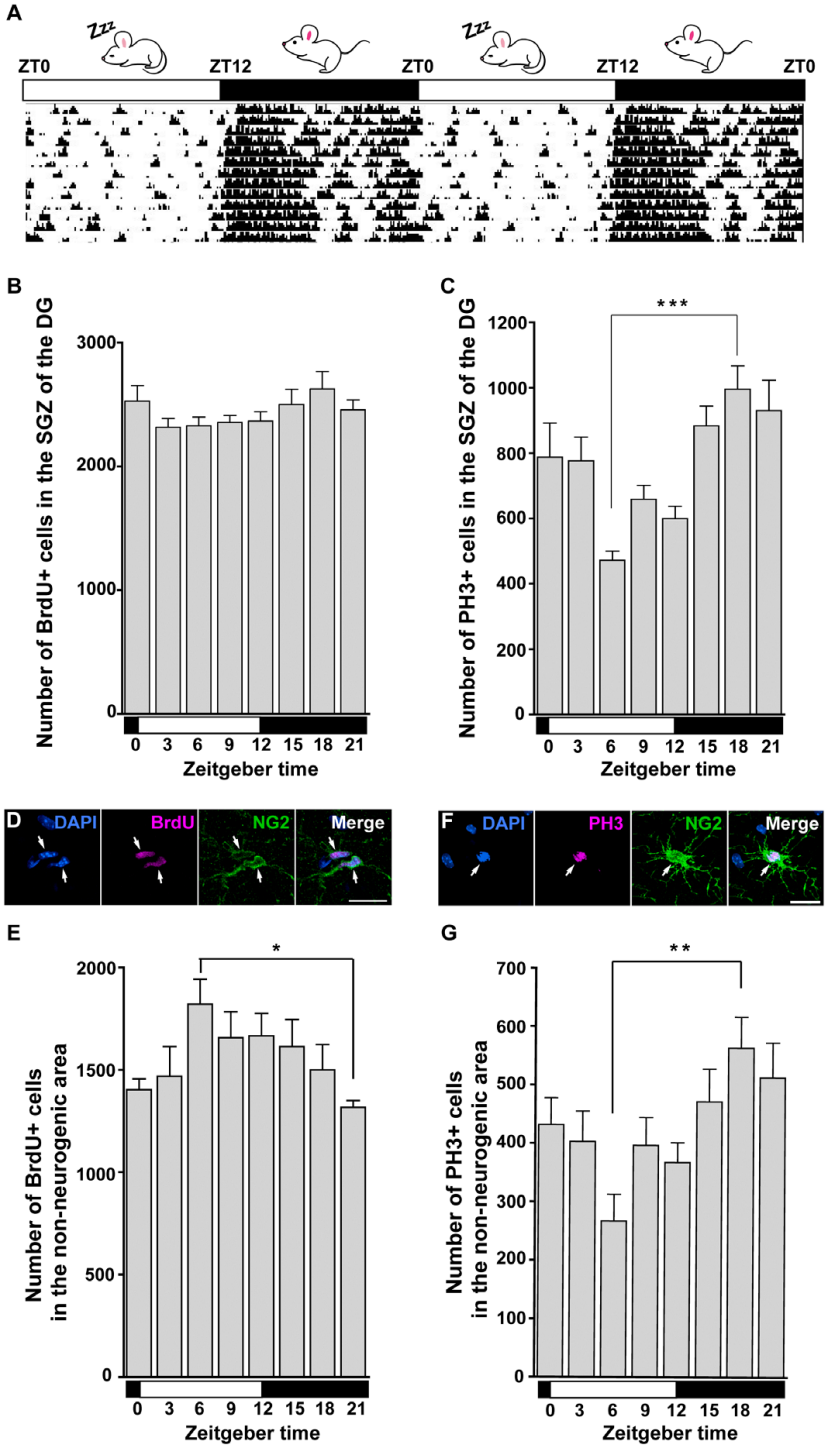
Daily variation in the number of BrdU-positive cells and PH3-positive cells in the neurogenic area and the non-neurogenic area of the hippocampus. (A) Representative actogram of the locomotor activity of a mouse reared under a constant L-D cycle. The white and black horizontal bars represent the light and dark periods, respectively, across two days. Black bars on each line represent the amount of locomotor activity. (B) Total number of BrdU-positive cells in the neurogenic area at various ZT (mean ± s.e.m., n = 8 to 10 for each time point). White and black bars represent light and dark periods of the day, respectively. There was no significant variation in the number of BrdU-positive cells between each time point ZT (*p* = 0.322 by one-way ANOVA). (C) Total number of PH3-positive cells in the neurogenic area at various ZT. The number of PH3-positive cells was the highest at ZT18 and the lowest at ZT6 (mean ± s.e.m., n = 8 for each time point, *p*<0.001 by one-way ANOVA, ***p<0.001 and by a post hoc Tukey test). (D) BrdU-positive cells labeled with NG2 in the non-neurogenic area. (E) Total number of BrdU-positive cells in the non-neurogenic area at various ZT (mean ± s.e.m., n = 8 to 10 for each time point). The number of BrdU-positive cells was the highest at ZT6 and the lowest at ZT21 (*p* = 0.021 by one-way ANOVA, *p<0.05 by a post hoc Tukey test). (F) PH3-positive cell labeled with NG2 in the non-neurogenic area. (G) Total number of PH3-positive cells in the non-neurogenic areas at various ZT. The number of PH3-positive cells was the highest at ZT18 and the lowest at ZT6 (mean ± s.e.m., n = 8 for each time point, *p* = 0.004 by one-way ANOVA, **p<0.01 by a post hoc Tukey test).

To evaluate the proliferation at distinct daily times, we injected BrdU into mice exhibiting a regular oscillatory pattern of locomotor activity (Figure 2A) under constant light-dark cycles (L-D; 12-hour light and 12-hour dark), at various zeitgeber times (ZT), and examined BrdU-positive cells that had passed through S-phase. To know the number of mitotic cells, the expression of phosphorylated histone H3 (PH3) was examined every 3 hours. The neurogenic area showed daily variations in the number of PH3-positive cells, i.e., a peak during nighttime (the active period), whereas the number of BrdU-positive cells seemed to be uniform (Figure 2B, C), as indicated in a previous report [17]. By contrast, we observed daily changes in the total numbers of BrdU-positive cells colocalized with NG2 (Figure 2D) in non-neurogenic area (Figure 2E). The number of BrdU-positive cells was significantly higher during daytime (the resting period) (ZT6: 1820±120 cells, n = 8) than during nighttime (ZT21: 1316±96 cells, n = 8; *p* = 0.040; Figure 2E), while the number of PH3-positive cells colocalized with NG2 (Figure 2F) was significantly higher during the nighttime (564±52 cells at ZT15, n = 8) than during the daytime (267±45 cells at ZT6, n = 8; *p* = 0.002; Figure 2G). Thus, within the non-neurogenic area, the daily changes in the number of PH3-positive cells showed an inverse correlation with the variation in the number of BrdU-labeled cells. Therefore, cells undergoing S-phase in the daytime are likely to progress through the cell cycle to M-phase in the nighttime. Collectively, these results suggest that OPCs show synchronized proliferation in the hippocampus and the proliferation peaks during nighttime. Differential regulation of cell-cycle progression in the neurogenic and non-neurogenic areas might be attributed to distinct styles of proliferation.

### Cyclin D1 is expressed in OPCs in the hippocampus

**Figure 3:**
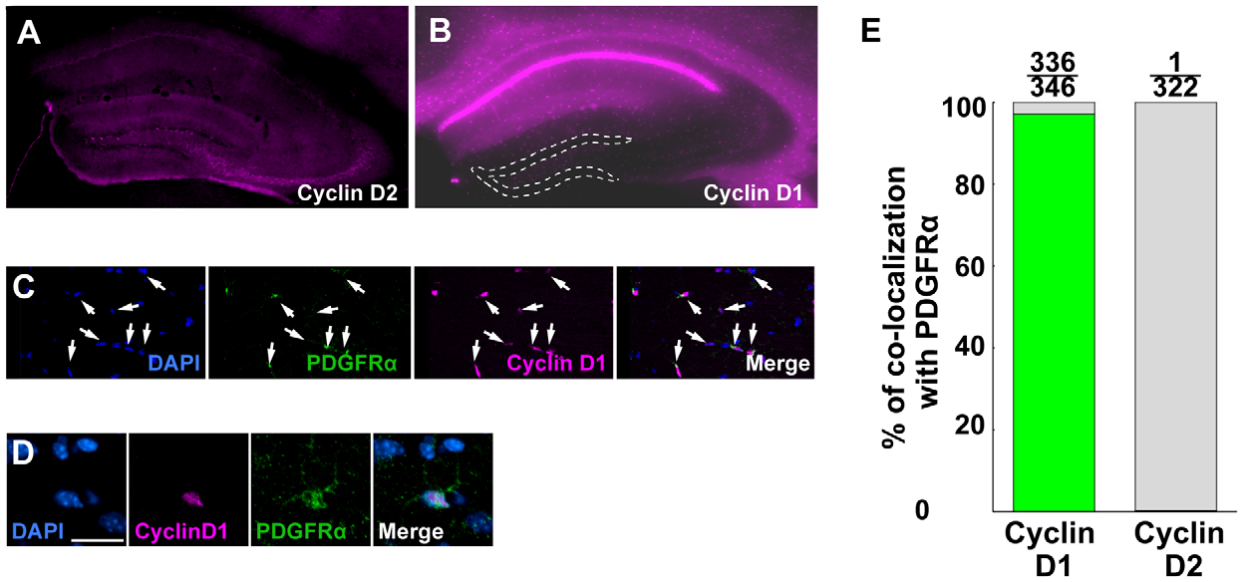
Cyclin D1 is expressed in OPCs in the hippocampus. (A) Immunostaining with anti-cyclin D2 antibody in the hippocamous. Immunoreactive signals were seen along the SGZ of the DG. (B) Immunostaining with anti-cyclin D1 antibody in the hippocampus. White dots show the margin of the DG. The bright band of immunoreactivity seen above the DG is the cluster of pyramidal neurons in CA1 as previously reported [47]. (C and D) Double immunostaining with anti-PDGFRα and anti-cyclin D1 antibodies in the hippocampus. White arrows indicate PDGFRα-positive cells that are positive for cyclin D1. Higher magnitude image of double positive cell (D). Scale bar: 20 µm. (E) The percentage of cells positive for each marker among all PDGFRα-positive cells.

Cyclin D family are cell-cycle regulatory proteins that control cell cycle progression from G1 to S-phase. A previous study revealed that proliferation of neural progenitor cells is controlled by cyclin D2 in SGZ [20]. We reproducibly observed that cyclin D2 positive cells were located along the SGZ of the DG (Figure 3A). Contrastingly, cyclin D1 positive cells showed a different distribution pattern with scattered expression in CA1 to CA3 regions in the hippocampus (Figure 3B). We performed double immunostaining with cyclin D1 or cyclin D2 along with PDGFRα, and observed that cyclin D1 was expressed in PDGFRα-positive cells in the entire hippocampus (97.1%: n = 4, Figure 3C, D, E), while cyclin D2 only showed a negligible level of expression in the PDGFRα-positive cells (Figure 3E). It is thus speculated that OPC proliferation might be regulated by cyclin D1, while cyclin D2 controls neurogenesis.

### OPCs enhance their proliferation by voluntary exercise

Several studies have shown that voluntary exercise increases cell proliferation in the hippocampal neurogenic area [21], [22], [23]. Therefore, we tested whether the exercise could similarly affect OPC proliferation. Mice were maintained under L-D cycles for one week and then allowed to exercise by voluntary wheel-running for an additional week. The actogram shows a representative wheel-running activity of a mouse reared under L-D cycles (Figure S2A). Wheel-running was observed mostly during the nighttime, in accordance with nocturnal activity. We analyzed the number of proliferating cells by injecting BrdU at eight-hour intervals for one day at the final day. As previously reported, increase of the number of BrdU-positive cells was seen in the neurogenic area of the running mice (12,453±976 cells in runners, 6,956±345 cells in non-runners, n = 8 in each group; *p*<0.001; Figure S2B). In the non-neurogenic area, the majority of BrdU-positive cells were labeled with PDGFRα in runners (90.1%, n = 4, Figure S2C, D), a result comparable to that in non-runners (90.8%, n = 4, Figure S2D). In this area, there was also a significant increase in the total number of BrdU-positive cells in the runners (4,015±179 cells, n = 8, Figure S2E) compared with the non-runners (2,750±59 cells, n = 8, *p*<0.001, Figure S2E). It is reported that voluntary wheel-running exercises can increase neuronal activity in the hippocampus [24].We indeed found a significant increase in the number of cells positive for c-Fos protein, an immediate early gene product up-regulated by neural activity [25], in the hippocampus of the running mice (9,983±1,003 cells, n = 4, Figure S3A, C), compared with the non-running mice (3,762±202 cells, n = 4, *p* = 0.005, Figure S3B, C). Taken together, voluntary exercise enhanced neuronal activity and increased the proliferation of OPCs in the hippocampus.

## Discussion

In the present study, we revealed for the first time that the majority of proliferating cells in the hippocampal non-neurogenic area are OPCs. The OPC is defined by its special morphology and associated molecular profiles [26]. Either Olig2, PDGFRα or NG2 is commonly used to identify OPCs, although each molecule is known to be expressed in other types of cells in the brain as well. For instance, Olig2 is expressed in neural stem cells and myelinating oligodendrocytes [9], PDGFRα in endothelial cells and mural cells [27], and NG2 in pericytes [28]. Thus, in this study, a combination of cell-type specific markers, Olig2, PDGFRα and NG2, was used to identify the proliferation characteristic of OPCs. Approximately 90% of BrdU-positive cells were positive for Olig2, PDGFRα and NG2, thus being defined as OPCs. This is consistent with previous studies showing that continuous proliferation of OPCs is evident throughout an adult mammalian brain [3], [5], [29], [30].

A previous study has shown that the day/night variations in the kinetics of S-phase cells in the hilus of the DG [31], a part of the non-neurogenic area. We elucidated these variations further by showing that peaks of S-phase and M-phase numbers of OPCs in the hippocampal non-neurogenic area were inversely correlated in a time-dependent manner. This may imply that the population undergoing S-phase during the daytime undergoes M-phase during the nighttime. By contrast, in the SGZ we showed a daily variation in the number of M-phase cells, but not in the number of S-phase cells, consistent with the previous study [17]. Thus proliferating cells in the neurogenic area proceeded with S-phase irrespective of the time of day, whereas cell cycle progression into M-phase could be suppressed until nighttime [17].

The difference in the mode of proliferation between OPCs and neural stem/progenitor cells in adult hippocampus is our novel finding and very intriguing. In this regard, it is of note that cyclin D1 was expressed in PDGFRα-positive OPCs in the hippocampus, while neural stem/progenitor cells in the SGZ did not express cyclin D1, but they expressed cyclin D2 as previously reported [20]. To our knowledge, OPC cultures showed that cyclin D1 plays a part in proceeding cell cycle in OPCs [32], [33], [34], and in *cyclin D2* knockout mice, hippocampal neurogenesis is dramatically reduced [20]. Differential regulation of cell-cycle progression of the neural stem cells and OPCs might be attributed to distinct types of cell-cycle regulatory proteins resided in proliferating cells. Since *cyclin D1* is regulated by *period 2* gene, one of the key circadian rhythm regulators [35], we thus assume that OPC proliferation might be regulated by clock gene(s). It would be interesting to know whether proliferation of OPCs is disturbed in mice deficient with clock genes.

Various stimuli can influence progenitor proliferation in adult rodent brains. Voluntary wheel-running exercise and learning tasks are reproducibly reported to increase neurogenesis in the rodent hippocampal DG [21], [36]. Here we show that voluntary wheel-running also enhances OPC proliferation in the mouse hippocampus. Another study has recently reported that in the grey matter of the cerebral cortex, voluntary physical exercise for 14 days decreased the number of active proliferating cells with increased exit of the cell cycle followed by enhanced differentiation into mature oligodendrocytes [37]. Discrepancies between our finding and this previous one might be attributed to the different areas or the different time course of the experiments. We focused on the hippocampus that shows the unique structure and function different from those of the cortex, and counted the BrdU-positive cells on the 7^th^ day of the running course so that we might be seeing halfway along the path of their destiny. Considering that increased expression of c-Fos in the hippocampus of the exercised mice reflects neuronal activity, it is possible that OPC proliferation is dependent on neuronal activity. We further assume that daily neural activity may influence on the proliferation of OPCs and/or that daily oscillation of OPC proliferation may directly or indirectly modulate neuronal functions in the hippocampus (see below).

Recent studies have shown that OPCs give rise to mature oligodendrocytes, which contribute to axonal myelination [38], [39], and to mature neurons in the piriform cortex in the adult rodent brain [39]. We observed existence of OPCs within the terminal fields of the mossy fibers, i.e., unmyelinated axons in the hippocampus [40] (Figure S4). It is thus curious what these OPCs in the non-neurogenic area of the hippocampus are doing homeostatically. OPC may serve dual roles, one as a source of myelinating oligodendrocytes and the other as that fulfilling some homeostatic functional role in the adult brain [41]. In the healthy normal brain, neural stem cells in the SGZ give rise to transiently amplifying progenitor cells, go through postmitotic stage [42], and become functional neurons in 4 to 7 weeks [43], whereas dividing OPCs can maintain action potentials, receive functional synaptic inputs, release synapse-modulatory substances and renew themselves as pairs of daughter cells in the hippocampus [11], [12]. Therefore, oscillatory proliferation of OPCs might have great impact on hippocampal function. For example, OPC proliferation itself could facilitate remodeling of neural circuits. Since chondroitin sulfate proteoglycans, components of an OPC marker NG2, play a role in learning and memory of the hippocampus [44], we predict that the proliferation of OPCs in response to neuronal activity may eventually modulate the synaptic plasticity for the hippocampal function. These ideas are warranted to be addressed in the future.

## Materials and Methods

### Ethics Statement

The protocols used for all animal experiments in this study were approved by the Animal Research Committee of Tohoku University (20MA-182).

### Housing conditions

Six-week-old male mice (C57BL/6J, Japan Charles River Inc.) were housed under 12-hour light (LED, 300 lux)/12-hour dark (L-D) cycles for two weeks to synchronize the phase of their internal clocks, with water and food available *ad libitum*. To monitor general motor activity, animals were kept individually in cages equipped with infrared motion detectors (ClockLab, Actimetrics Inc.) under the L-D cycles. The data were analyzed using MATLAB (The MathWorks Inc.), and activity records were plotted as actograms. The time of day is designated as zeitgeber time (ZT). ZT0 and ZT12 correspond to the times when the light was turned on and off during the L-D cycles, respectively.

### BrdU injection

To assess daily changes in cell proliferation, animals were intraperitoneally injected with 50 mg/kg of 5-bromo-2′-deoxyuridine (BrdU, Sigma-Aldrich). Every three hours throughout the L-D, a mouse was given a single injection and sacrificed three hours afterward. To identify proliferating cell types and to assess the effect of physical exercise on cell proliferation, animals were intraperitoneally injected with BrdU (50 mg/kg) three times at ZT0, ZT8 and ZT16 and then sacrificed at the following ZT0.

### Immunohistochemistry

Immunohistochemistry (IHC) analysis of cryosections was performed as described in previous reports [45], [46]. Briefly, frozen coronal sections (16 µm thickness) were prepared through the entire rostrocaudal extent of the hippocampus using a cryostat (CM3050, Leica Instruments), and every fourth section was selected for IHC. To quantify the number of BrdU-labeled cells, every fourth section of the entire hippocampus was observed using a confocal laser scanning microscope (LSM-PASCAL, Zeiss), and the total number of positive cells was multiplied by four. For the quantification of c-Fos, every sixth section taken at ZT18 was observed using a fluorescence microscope (BIOREVO, Keyence) and processed for automated counting using the cell count software (BZ-H1C, Keyence), and the total number of positive cells was multiplied by six. To identify proliferating cell types, coronal brain slices (40 µm thickness) were made with a vibratome (Microslicer, Dosaka), and double-immunofluorescent labeling against BrdU and cell-type-specific markers was performed as described previously [46]. The primary antibodies used in this study are shown in Table S1. For secondary antibodies, we used DyLight488-, Cy3- or Cy5-conjugated anti-mouse, anti-rabbit or anti-rat antibodies (Jackson ImmunoResearch) and Alexa 488- or 555-conjugated anti-mouse or anti-rabbit antibodies (Invitrogen/Molecular Probes). More than 200 BrdU-positive cells from four animals were analyzed with a confocal laser scanning microscope to identify cells co-expressing BrdU and other cell-type-specific markers.

### Wheel-running exercise

Mice were housed individually in transparent plastic cages (35×20×20 cm). Each cage was equipped with a running wheel 15 cm in diameter, which turned a microswitch during each revolution. Wheel-running activity was continuously recorded in 5-min intervals by a data-logger system (CardBus TYPE II size PC Card for input/output of digital signals PIO-16/16L(CB)H, CONTEC CO., LTD). In the control group (non-runners), mice were individually kept in cages with locked running wheels. The mice were maintained under the L-D cycles for one week and then allowed to exercise by voluntary wheel-running for an additional week.

### Statistical analyses

Statistical analysis of the daily variation in the number of proliferating cells was done via one-way ANOVA followed by a *post hoc* Tukey test, and evaluation of statistical differences between runners and non-runners was performed using two-tailed Student's *t* tests (SPSS software, SPSS Inc.). *p*<0.05 was considered statistically significant.

## Supporting Information

Figure S1 **Schematic diagram showing the neurogenic area and the non-neurogenic area in mouse hippocampus.** SGZ, subgranular zone; GCL, granular cell layer; DG, dentate gyrus. The neurogenic area (SGZ, pink dotted line) is a narrow layer of cells bordered the GCL of the DG. The non-neurogenic area (light orange) includes entire hippocampal grey matter excluding SGZ.
(TIF)

Figure S2 **Increased neural progenitor/OPC proliferation in the hippocampus with wheel-running exercise.** (A) Representative actogram of a mouse performing wheel-running exercise under L-D cycles. (B) Total number of BrdU-positive cells in the non-runner and runner groups in the neurogenic area (mean ± s.e.m., n = 8, ***p<0.001 by two-tailed Student's *t*-test). (C) Expression of PDGFRα in BrdU-positive cells in exercised mice (white arrows). Scale bar: 20 µm. (D) The percentage of PDGFRα-positive cells among all BrdU-positive cells in non-runner and runner mice. (E) Total number of BrdU-positive cells in the non-runner and runner groups in the non-neurogenic area (mean ± s.e.m., n = 8, ***p<0.001 by two-tailed Student's *t*-test).
(TIF)

Figure S3 **Wheel-running exercise increases c-Fos expression in the hippocampus during the nighttime.** (A) DG stained with anti-c-Fos antibody in non-runners at ZT18. (B) DG stained with anti-c-Fos antibody in runners at ZT18. Scale bars: 50 µm. (C) Total number of c-Fos-positive cells in the hippocampus at ZT18 in non-running and running mice (mean ± s.e.m., n = 4 for each group, **p<0.01 by two-tailed Student's *t*-test).
(TIF)

Figure S4 **OPCs in the mossy fiber terminal field in the hippocampus.** (A) Schematic representation of the mossy fiber (MF) connections on the CA3 pyramidal neurons (referred to [48]). The dentate granule cells send unmyelinated axons to the CA3 region. (B) Double immunostaining with synaptophysin, a marker for synaptic vesicle protein, and PDGFRα of the black box in the scheme of hippocamous (A). Since MFs are known to course and form giant synaptic terminals [49], accumulation of synaptophysin immunoreactivity along with CA3 pyramidal cells (yellow dots) were seen [50]. PDGFRα were detected among the synaptophysin immunoreactivity, the MFs pathway. Scale bar: 20 µm.
(TIF)

Table S1 **Primary and secondary antibodies used in the experiments.**
(TIF)
