# Supplementary figures and images for: Differential Proliferation Rhythm of Neural Progenitor and Oligodendrocyte Precursor Cells in the Young Adult Hippocampus
Source: PLoS One. 2011 Nov 14;6(11):e27628. doi: 10.1371/journal.pone.0027628 (PMC3215740; doi:10.1371/journal.pone.0027628)

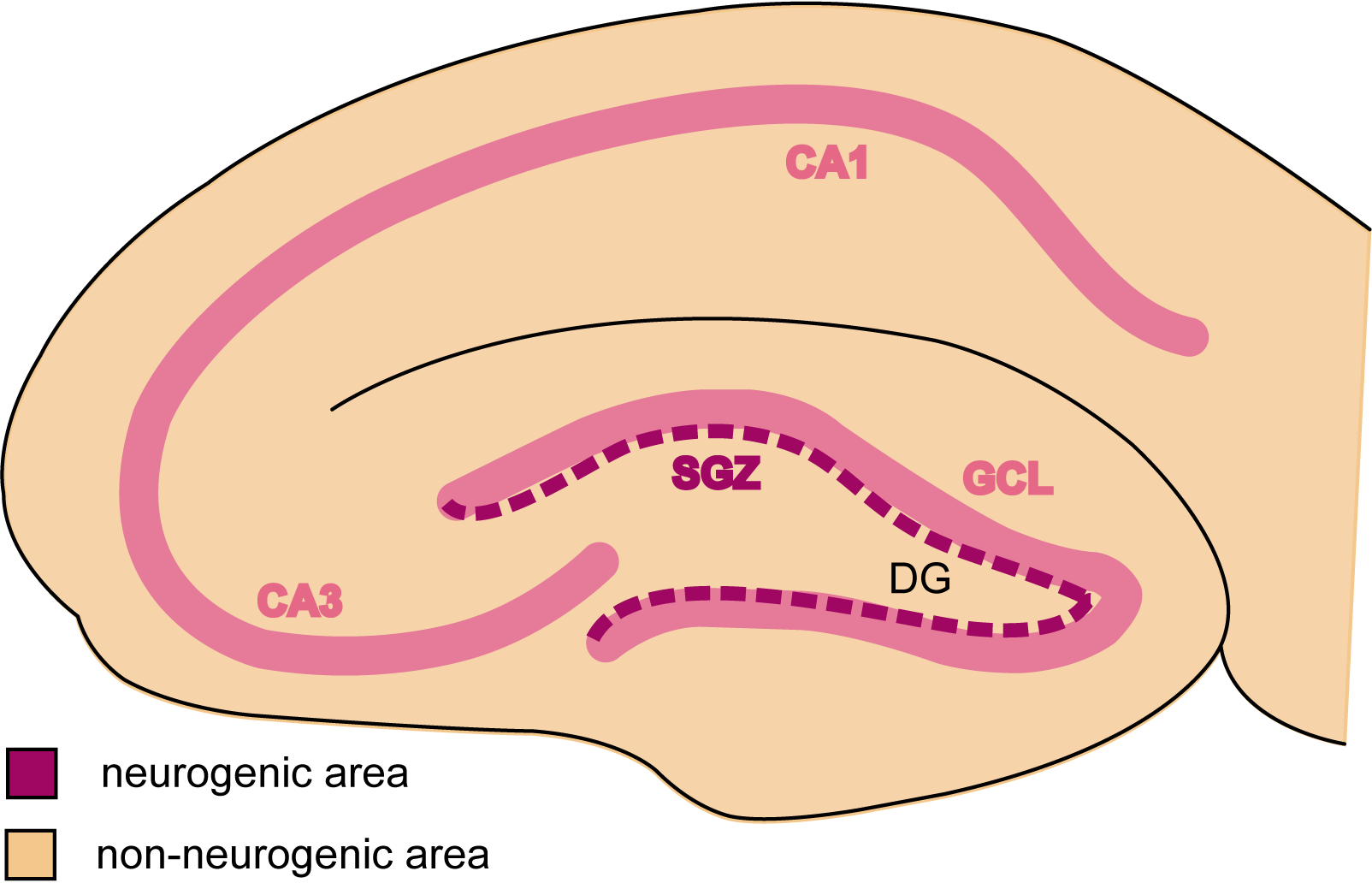

Supplement: Figure S1 — Schematic diagram showing the neurogenic area and the non-neurogenic area in mouse hippocampus. SGZ, subgranular zone; GCL, granular cell layer; DG, dentate gyrus. The neurogenic area (SGZ, pink dotted line) is a narrow layer of cells bordered the GCL of the DG. The non-neurogenic area (light orange) includes entire hippocampal grey matter excluding SGZ. (TIF) [file pone.0027628.s001.tif]

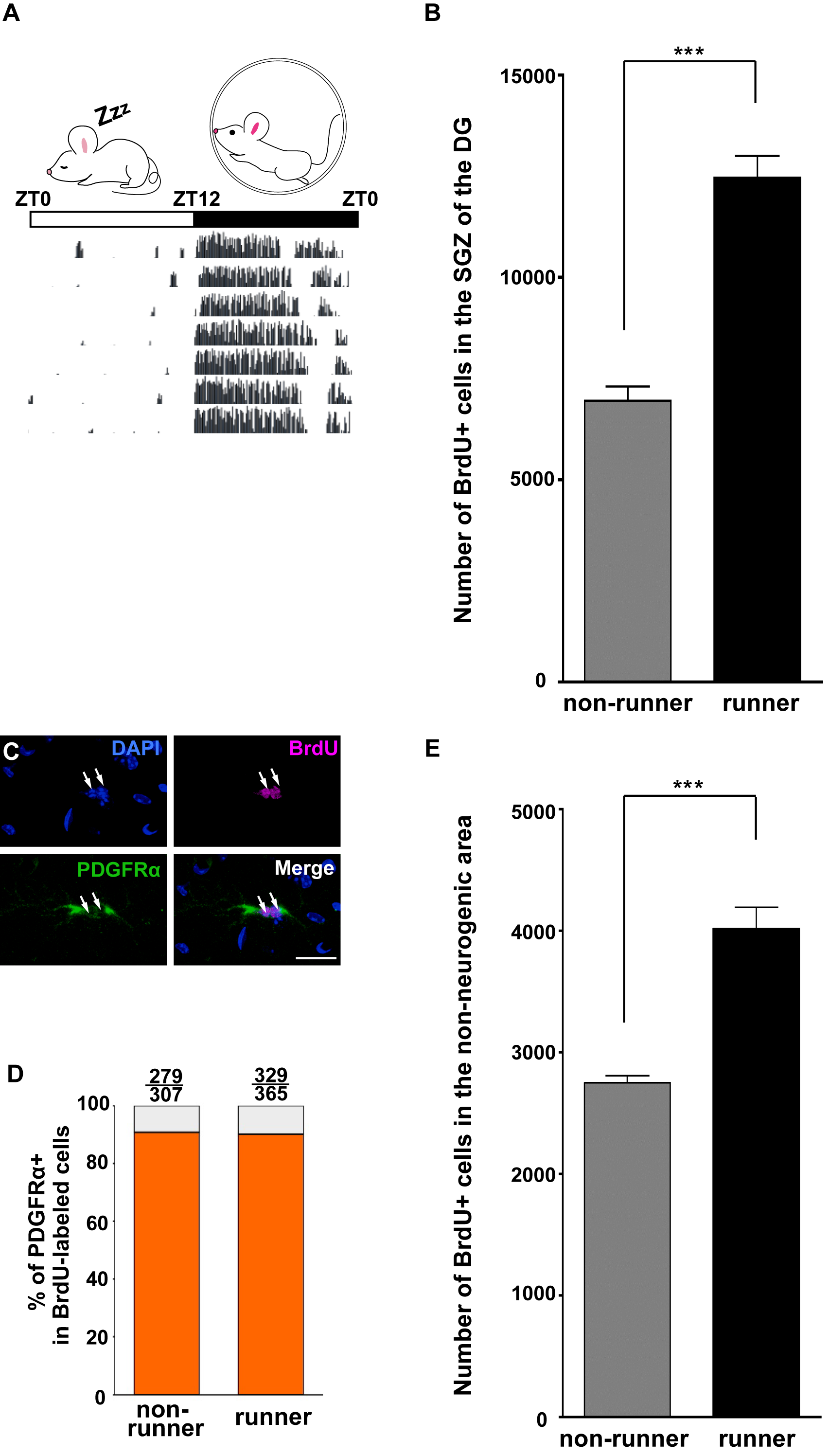

Supplement: Figure S2 — Increased neural progenitor/OPC proliferation in the hippocampus with wheel-running exercise. (A) Representative actogram of a mouse performing wheel-running exercise under L-D cycles. (B) Total number of BrdU-positive cells in the non-runner and runner groups in the neurogenic area (mean ± s.e.m., n = 8, ***p<0.001 by two-tailed Student's t-test). (C) Expression of PDGFRα in BrdU-positive cells in exercised mice (white arrows). Scale bar: 20 µm. (D) The percentage of PDGFRα-positive cells among all BrdU-positive cells in non-runner and runner mice. (E) Total number of BrdU-positive cells in the non-runner and runner groups in the non-neurogenic area (mean ± s.e.m., n = 8, ***p<0.001 by two-tailed Student's t-test). (TIF) [file pone.0027628.s002.tif]

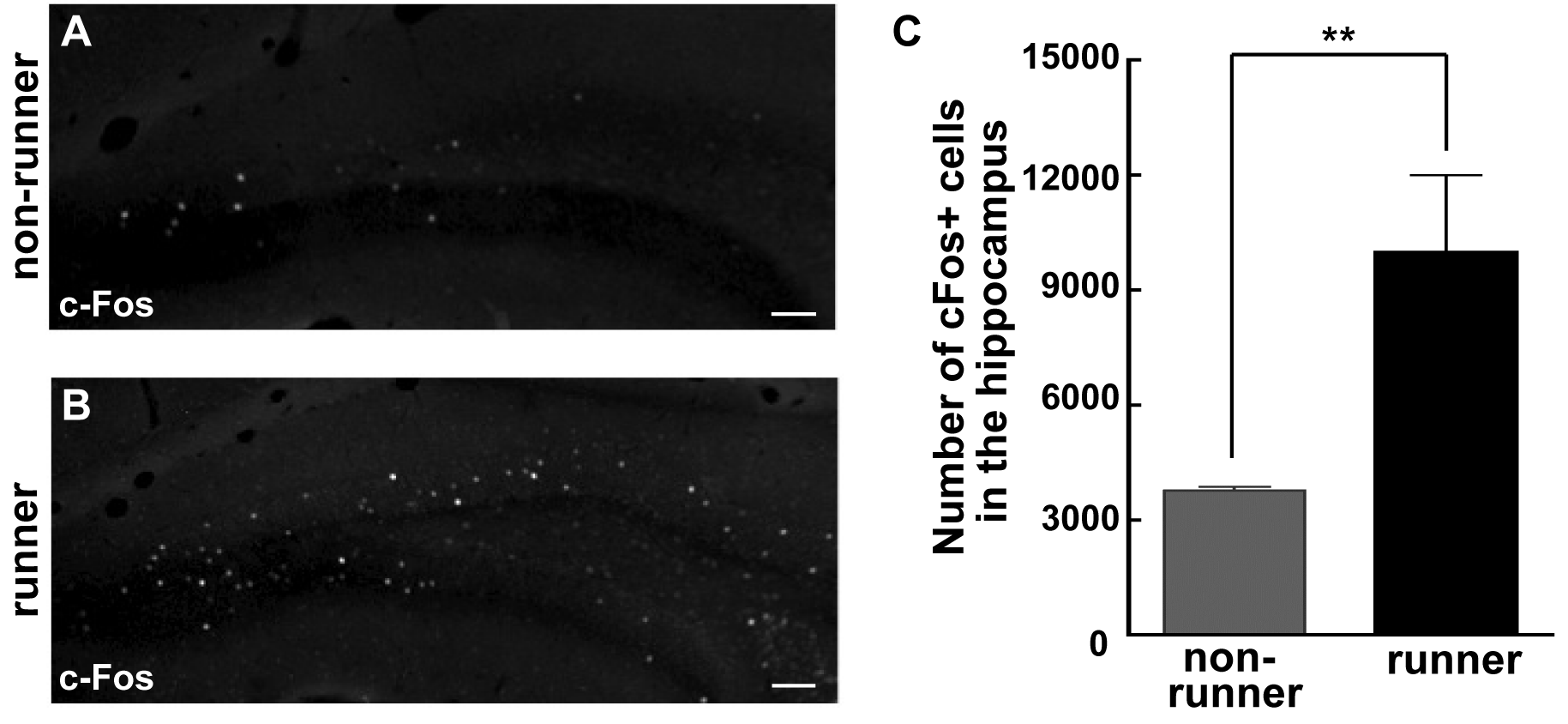

Supplement: Figure S3 — Wheel-running exercise increases c-Fos expression in the hippocampus during the nighttime. (A) DG stained with anti-c-Fos antibody in non-runners at ZT18. (B) DG stained with anti-c-Fos antibody in runners at ZT18. Scale bars: 50 µm. (C) Total number of c-Fos-positive cells in the hippocampus at ZT18 in non-running and running mice (mean ± s.e.m., n = 4 for each group, **p<0.01 by two-tailed Student's t-test). (TIF) [file pone.0027628.s003.tif]

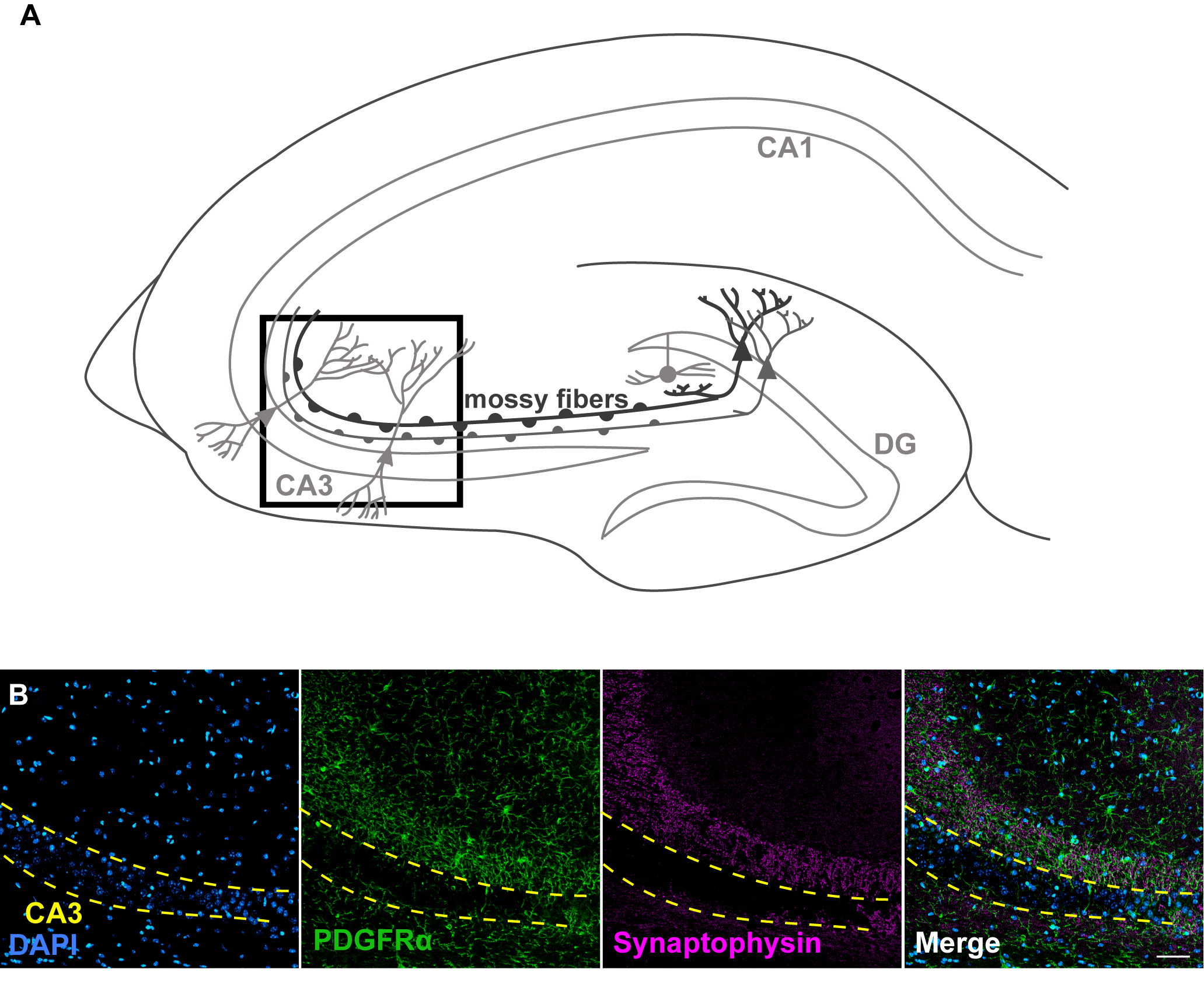

Supplement: Figure S4 — OPCs in the mossy fiber terminal field in the hippocampus. (A) Schematic representation of the mossy fiber (MF) connections on the CA3 pyramidal neurons (referred to [48]). The dentate granule cells send unmyelinated axons to the CA3 region. (B) Double immunostaining with synaptophysin, a marker for synaptic vesicle protein, and PDGFRα of the black box in the scheme of hippocamous (A). Since MFs are known to course and form giant synaptic terminals [49], accumulation of synaptophysin immunoreactivity along with CA3 pyramidal cells (yellow dots) were seen [50]. PDGFRα were detected among the synaptophysin immunoreactivity, the MFs pathway. Scale bar: 20 µm. (TIF) [file pone.0027628.s004.tif]

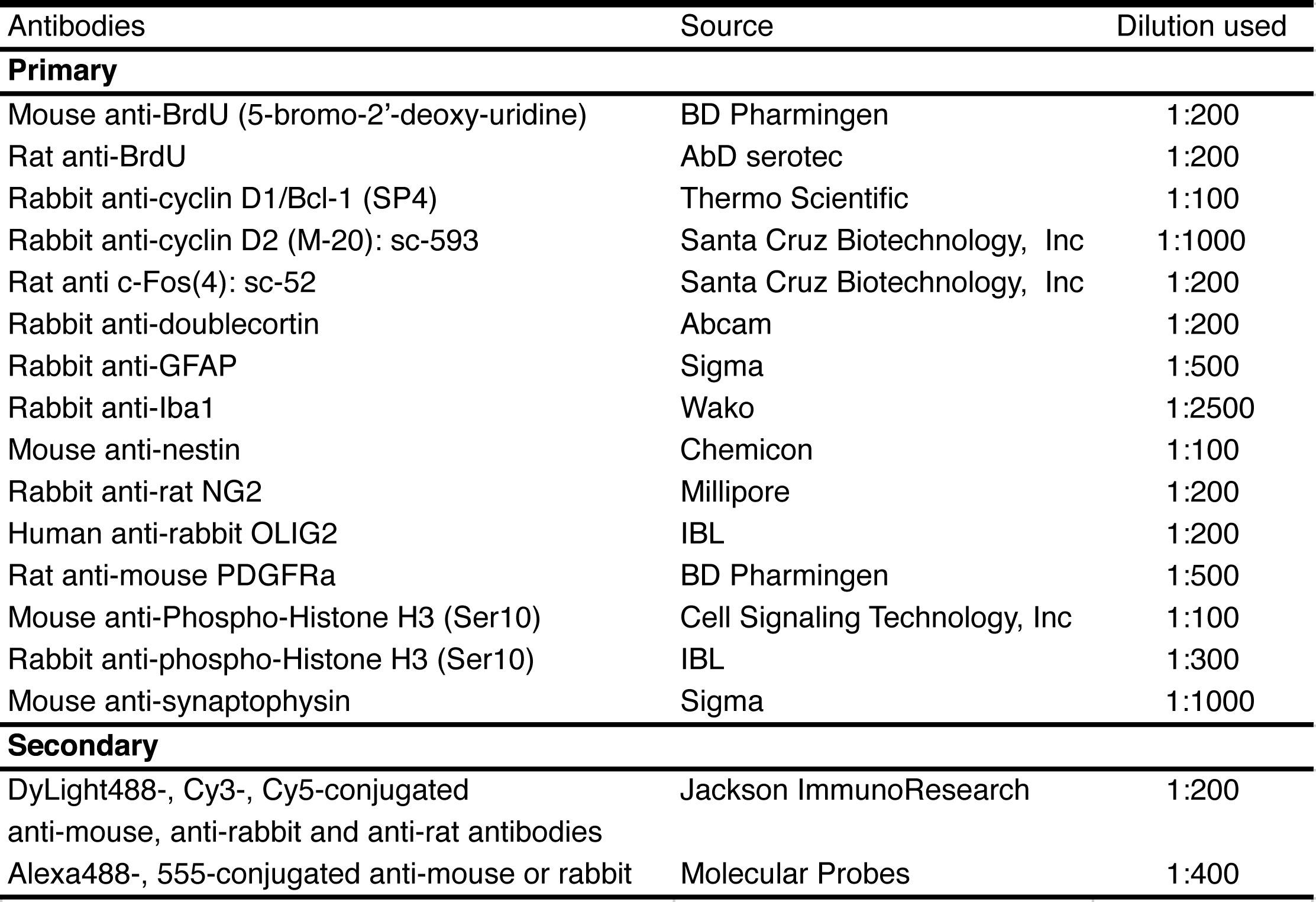

Supplement: Table S1 — Primary and secondary antibodies used in the experiments. (TIF) [file pone.0027628.s005.tif]
